# Retraction: Knowledge, attitude, practice towards COVID-19 pandemic and its prevalence among hospital visitors at Ataye district hospital, Northeast Ethiopia

**DOI:** 10.1371/journal.pone.0330161

**Published:** 2025-08-13

**Authors:** The PLOS One Editors

This article [1] was identified as one of a series of submissions for which we have concerns about authorship and adherence to the journal’s first and second publication criteria.

In addition, the editors have concerns about the integrity of the underlying data provided in post-publication discussions. These concerns were not resolved in discussions with the authors.

In light of these issues, the *PLOS One* Editors retract this article.

DG, EK, SG, and MAB did not agree with the retraction. NA and MA either did not respond directly or could not be reached.
